# Artificial Synapse: Spatiotemporal Heterogeneities in Dopamine Electrochemistry at a Carbon Fiber Ultramicroelectrode

**DOI:** 10.1021/acsmeasuresciau.1c00006

**Published:** 2021-07-01

**Authors:** Baoping Chen, David Perry, James Teahan, Ian J. McPherson, James Edmondson, Minkyung Kang, Dimitrios Valavanis, Bruno G. Frenguelli, Patrick R. Unwin

**Affiliations:** ^†^Department of Chemistry, ^‡^Molecular Analytical Science Centre for Doctoral Training, and ^§^School of Life Sciences, University of Warwick, Coventry, CV4 7AL, United Kingdom

**Keywords:** Scanning Ion Conductance Microscopy (SICM), Electrochemical Imaging, Exocytosis, Nanopipettes, Single Entity Electrochemistry

## Abstract

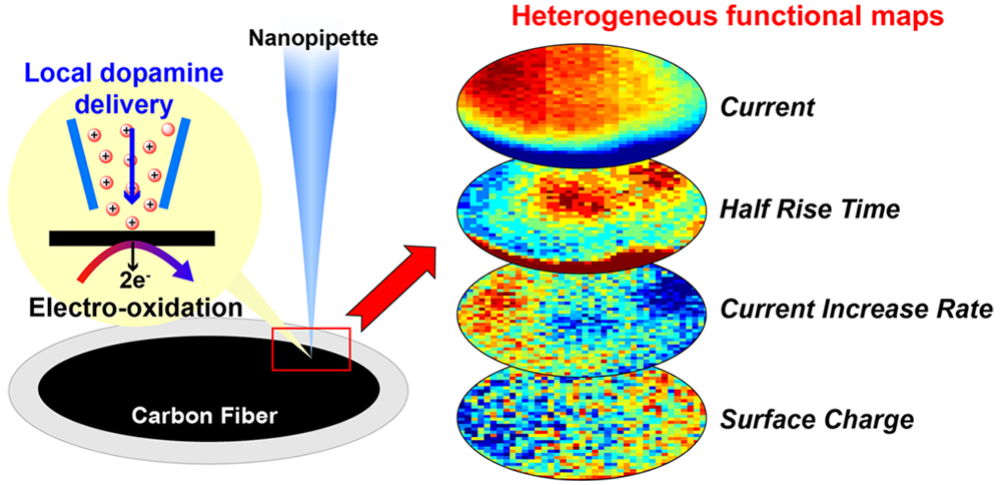

An artificial synapse is developed that mimics ultramicroelectrode (UME) amperometric detection of single cell exocytosis. It comprises the nanopipette of a scanning ion conductance microscope (SICM), which delivers rapid pulses of neurotransmitter (dopamine) locally and on demand at >1000 defined locations of a carbon fiber (CF) UME in each experiment. Analysis of the resulting UME current-space-time data reveals spatiotemporal heterogeneous electrode activity on the nanoscale and submillisecond time scale for dopamine electrooxidation at typical UME detection potentials. Through complementary surface charge mapping and finite element method (FEM) simulations, these previously unseen variations in electrochemical activity are related to heterogeneities in the surface chemistry of the CF UME.

Synaptic signal transmission is the primary mechanism of cell to cell communication in the nervous system, for which vesicular exocytosis from an emitting cell is a key process.^1^ Exocytosis involves (part) fusion of a vesicle with the inside of the emitting cell membrane to create a fusion pore from which the vesicle contents are released.^2,3^ Mechanistic aspects of vesicular release have been studied by using a carbon fiber (CF) ultramicroelectrode (UME), positioned close to a target single cell, to monitor exocytotic events upon cell stimulation^4−8^ via chronoamperometric (current–time) detection of electroactive neurotransmitters via electrooxidation. This configuration results in highly localized transient electrochemical detection at the UME because the vesicular sources are tens to hundreds of nanometers in diameter, with the size depending on the neuron type.^7^ Herein, we introduce a scanning ion conductance microscopy (SICM) system that enables the delivery of rapid pulses of dopamine transiently and locally, at thousands of defined locations at a CF UME, mimicking exocytosis cell release-UME detection. The electrochemical signatures are analyzed and related to the nanoscale electrode surface properties *at the locations where the responses are measured*. This allows us to determine whether local electrode surface properties have any bearing on the chronoamperometric response at a CF UME.

SICM is a noncontact scanning probe microscopy technique that employs a nanopipette tip, enabling multifunctional mapping of a wide range of surface properties.^9−12^ For this work, we used single-barrel nanopipette tips (∼100 nm diameter; SI, Figure S1), filled with an aqueous solution of 100 mM dopamine hydrochloride (pH 5.8) of the same order of concentration as in a vesicle,^13,14^ whose contents could be released and collected on demand at a CF UME (∼7 μm diameter) surface. This configuration creates an artificial synapse^15^ that mimics the time scale and spatial dimension of a single cell synaptic release measurement (Figures 1 and Figure S2). HEPES physiological saline, containing 150 mM NaCl and 10 mM HEPES (pH 7.4), was used as the (bulk) electrolyte, which bathed the CF UME. Two Ag/AgCl electrodes were used as quasi-reference counter electrodes (QRCEs), one in the bulk solution (QRCE_bulk_), and the other inside the tip (QRCE_tip_). With the CF UME (working electrode) at ground, adjustment of the QRCE_bulk_ potential versus ground served to control the CF UME potential with respect to QRCE_bulk_. Further details on the experiments, including equilibrium potentials of the two QRCEs and the electrochemical setup, are provided in SI-1 and SI-2.

**Figure 1:**
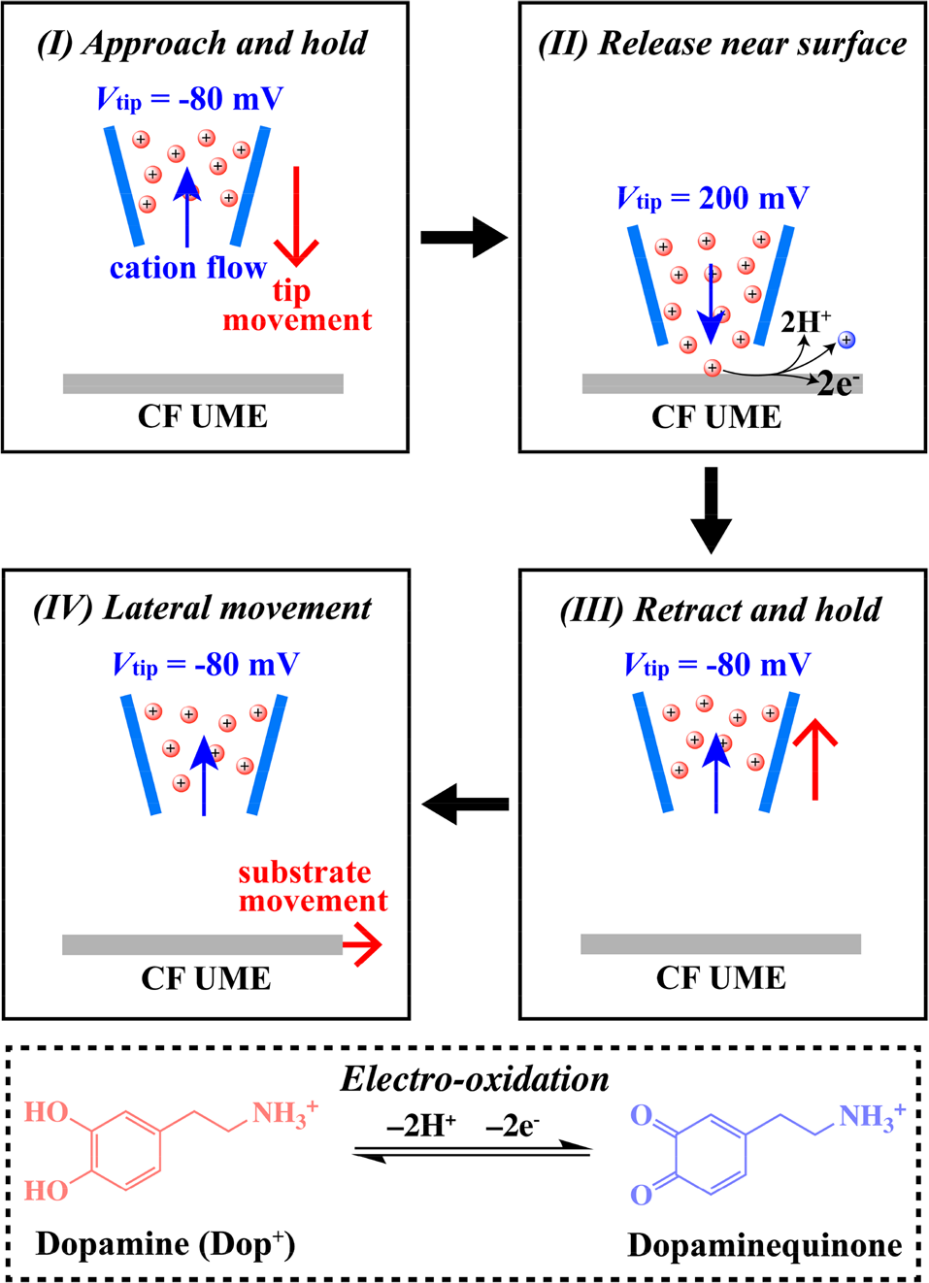
Schematic of the main features of the SICM hopping-potential pulse protocol, illustrating the translation of the tip and changes in the applied potential to enable the controlled release of Dop^+^ at a single pixel (described in the text). The procedure was repeated >1000 times in fresh locations across a predefined grid over the UME. The inset schematic illustrates the major dopamine electrooxidation process.

Electrode mapping utilized a hopping-potential pulse mode of SICM, with the protocol for a single pixel illustrated in Figure 1. (I) The tip was translated toward the UME substrate with QRCE_tip_ biased at −80 mV with respect to QRCE_bulk_ to produce an ionic current that was sensitive to the vertical position of the tip near the UME surface,^16^ while holding the protonated dopamine (Dop^+^) in the tip.^17^ At this small potential bias, the SICM response is primarily sensitive to tip–substrate distance.^18^ (II) When the tip reached the near surface, Dop^+^ was released by stepping *V*_tip_ to 200 mV versus QRCE_bulk_ for 20 ms. (III) Dop^+^ release was terminated by stepping *V*_tip_ back to −80 mV, as the tip was simultaneously retracted to the bulk. (IV) After 200 ms to allow re-establishment of initial conditions,^19^ the UME was moved laterally and the same procedure was executed at the next (fresh) point on the surface. The UME was biased at 0.7 V throughout (relative to QRCE_bulk_), at the diffusion-limit for electrooxidation of Dop^+^ as determined by voltammetry at the entire UME (see SI-3), and typical of that applied in amperometric monitoring of exocytosis.^7,8^ Both the tip and substrate currents were measured continuously throughout.

For SICM mapping, the tip (∼100 nm diameter) was approached to a working distance of ∼37 nm, as estimated from finite element method (FEM) simulations (see SI-6), for a decrease in the tip current magnitude by 2% from the bulk value at each approach. We are interested in situations where the nanopipette tip is directly over the CF surface to mimic the detection of exocytotic release, and exemplar data cropped to the central ∼6 μm diameter of the CF (to avoid complications from edge effects) are shown in Figure 2 in several different forms. Figure 2a shows 3 example substrate current–time (*I*_sub_-*t*) transients, at different locations of the CF UME (marked in Figure 2b). The *I*_sub_-*t* curves have the same general shape, that is, *I*_sub_ rises to a quasi-steady value after a short delay, but there are differences in the magnitude of *I*_sub_.

**Figure 2:**
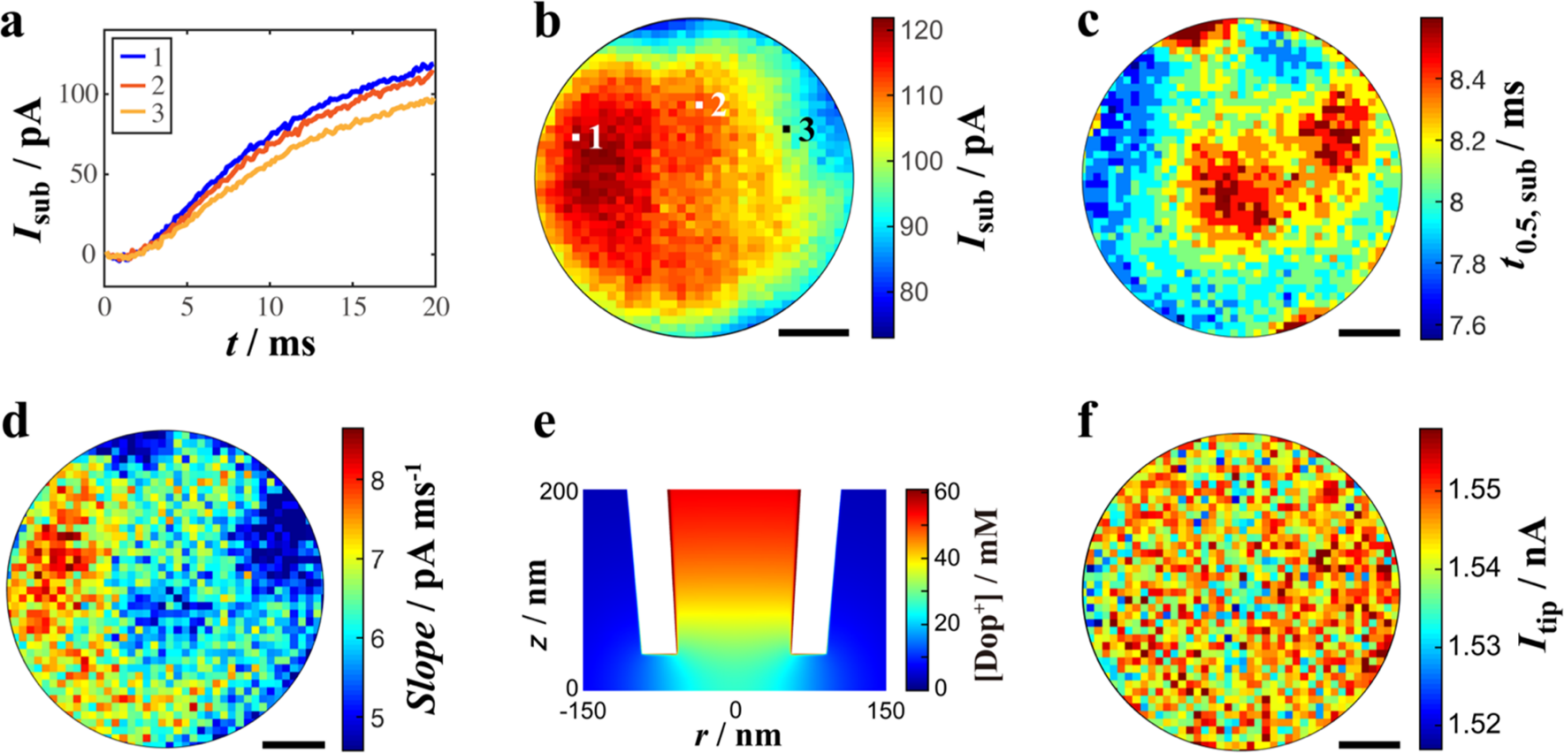
(a) Three typical *I*_sub_-*t* transients at pixels marked in (b). Images across the central 6 μm diameter of a CF UME of (b) final value of *I*_sub_ for each release pulse, (c) time for *I*_sub_ to reach half the final value, (d) rate of increase of *I*_sub_ at a time of 2.5 ms after the pulse, and (f) *I*_tip_ at the end of potential pulse. (e) Typical simulated concentration profile for Dop^+^ at the end of pulse release, with a rate of electrooxidation commensurate with the experimentally observed UME current values, in this case a current of 88 pA at the end of the Dop^+^ release pulse (see SI, Figure S12). Step size between pixels: 150 nm, with no interpolation of data. Scale bar: 1.2 μm.

The extent to which the current response is heterogeneous across the UME substrate is evident from Figure 2b, which shows a map of the final *I*_sub_ for each pulse release; the current varies by ∼33% (minimum to maximum value). Figure 2c further highlights heterogeneous activity in the time dimension, showing sub-millisecond variations in the time for *I*_sub_ to reach half the final value, while Figure 2d maps the rate of increase of *I*_sub_ at a time of 2.5 ms after the Dop^+^ pulse, again highlighting spatiotemporal variations in electrode activity. The spatial resolution is time-dependent, as evident from Movie S1 (see SI-8, cf., 4 ms where strongly localized activity is evident with 10 ms where there is still heterogeneous activity, but a radial component due to Dop^+^ lateral diffusion emerges in the background current). This is also seen when comparing Figure 2d (at 2.5 ms) with Figure 2b (at 20 ms), albeit for different activity signatures. This is consistent with the simulated concentration profile for Dop^+^ undergoing oxidation at the UME surface at a typical current of 88 pA (Figure S12). While the Dop^+^ detection potential of the CF UME was set to mimic exocytosis-UME detection protocols^7,8,20^ and is in the diffusion-limited region based on the bulk voltammogram for 1 mM Dop^+^ in SI (Figure S4), the reaction is not diffusion-limited for nanoscale delivery-detection; the near interface concentration of Dop^+^ is finite, ∼20 mM in the region of the CF UME directly under the center of the nanopipette. Kinetic limitations are manifest as a significant anodic shift of Dop^+^ electrooxidation potential for adsorbed Dop^+^ at short time scales,^21^ and an anodic shift of the electrooxidation potential under exocytosis-UME detection measurements might further be expected due to the high Dop^+^ concentration oxidized locally and the consequent high local concentration of protons released (given the comparatively low buffer concentration herein and in typical exocytosis-UME measurements).^2−4,6,8,15^

To further highlight the reliability of these measurements, the *I*_sub_-*t* profile measured in our experiments is reproduced well in simulations, with a simple electrooxidation rate boundary condition (Dop^+^ flux) as the only adjustable parameter, as detailed in SI-7, and taking account of the RC time constant of the CF UME-artificial synapse.^22,23^ Importantly, the tip current (*I*_tip_) at the end of the pulse potential period is consistent at each pixel, varying by just a few percent from minimum to maximum across ∼1200 positions at all times (Figure 2f, Movie S2). This confirms the stability and consistency of the SICM delivery process, which is also evidenced by the narrow distribution of half time (1.25 ± 0.03 ms) for the tip release process in Figure S3c, defined as the time for *I*_tip_ to attain 50% of the final magnitude change. These results prove that the observed variations in the electrochemical response of the CF UME are due to heterogeneous electrode activity. Typical tip and substrate current–time behavior and substrate topography over the UME and surrounding glass are shown in Figure S3.

We now consider the origin of the heterogeneities in spatiotemporal electrochemical activity at the CF UME. Correlative electrochemical imaging–Raman microscopy has recently been used to analyze variations in dopamine electrooxidation at screen printed carbon electrodes,^24^ but the spatial variations in electrochemical activity observed in Figure 2 are beyond the diffraction limit. A qualitative indicator of variations in surface chemistry of the CF UME can be seen from contrast variations in field emission-scanning electron microscopy (FE-SEM) images of a typical CF UME surface (Figure S5); there is less charging (darker contrast) for more conductive regions and vice versa.^25^ These spatial heterogeneities occur on the several hundred nanometer scale, similar to the spatial variations in CF UME current for Dop^+^ electrooxidation.

To understand how electrode surface chemistry could influence the Dop^+^ electrooxidation current signal, we used SICM to map the surface charge of the CF UME (see SI-5),^18^ and the result was compared directly with the corresponding co-located electrochemical activity. Surface charge data were obtained in a separate scan just before the electrochemical activity mapping. The coalignment of electrode activity and surface charge maps is detailed in Figure S7. A surface charge map of the CF UME surface in the region of interest (extracted from the data in Figure S7), at a CF UME bias of *V*_sub_ = 0.7 V, as used for activity mapping, is shown in Figure 3a. There are significant surface charge heterogeneities across the CF surface. There is predominantly a negative surface charge density at the carbon electrode surface,^26^ attributed to the prevalence of surface oxygen-containing moieties on carbon electrodes, for example, surface oxides^27^ and surface carboxylates.^28−30^ Dop^+^ is considered to adsorb to these groups,^31^ and, even without adsorption, would be a significant component of the charge-compensating double layer under the experimental conditions. At least in part, the higher concentrations of Dop^+^ in these locations and the fact that adsorbed Dop^+^ may catalyze the oxidation of solution-phase Dop^+^^32^ explains the plot of *I*_sub_ versus CF UME local surface charge density in Figure 3b, where higher electrochemical currents are generally obtained in regions with more negative electrode surface charge. Surface roughness at the nanoscale and the nature of the resulting surface sites exposed^33^ will also be important for Dop^+^ electrooxidation kinetics, and Dop^+^ adsorption.^21^

**Figure 3:**
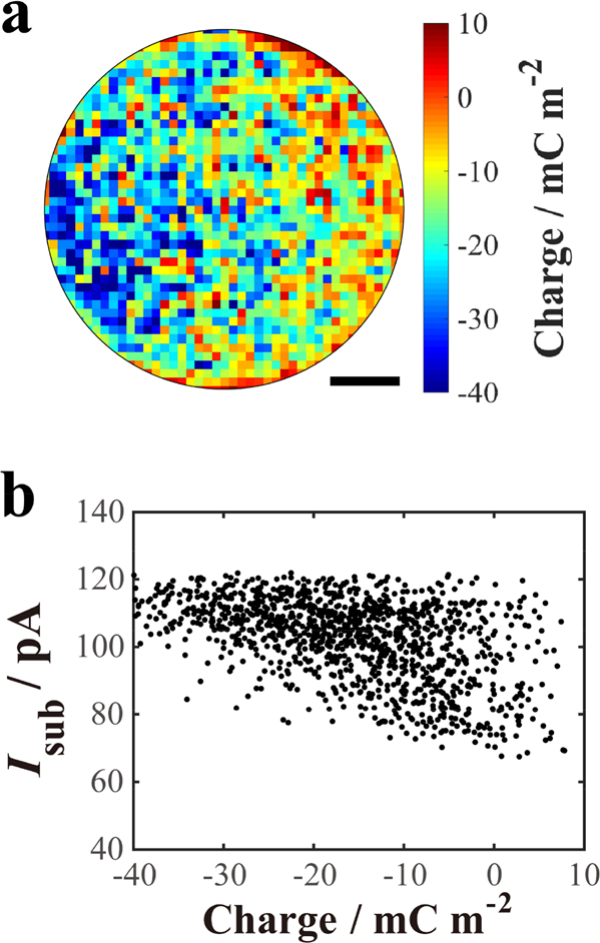
(a) Image of quantified surface charge in the same area of the CF UME as the electrochemical maps in Figure 2. Scale bar: 1.2 μm. (b) Correlation between *I*_sub_ and local surface charge at the CF UME surface.

In conclusion, this study reveals spatiotemporal variations in the rate of dopamine electrooxidation across a CF UME surface under conditions that mimic the amperometric detection of single cell exocytosis. Analysis of single cell exocytosis often involves the measurement of peak rise time (related to the opening kinetics of the fusion pore) and the peak (spike) half-width, which is indicative of the length of the duration event.^7^Figure 2c is a proxy for such measurements, and the overall variation between different electrode locations is on the sub-millisecond time scale (Figure 2). This is significant because exocytosis measurements usually report 1 ms (or longer) time resolution.^34,35^ Heterogeneity in activity becomes a more important consideration for faster measurements, where detection is more localized (less lateral diffusion to neighboring sites on the electrode), although there maybe scope for using higher oxidation potentials to push detection closer to the diffusion limit, being mindful of the onset of the anodic oxidation of water and the CF UME.

## Supporting Information Available

Typical STEM image of the nanopipettes used in this work, details about the experiments, voltammetric and FE-SEM characterizations of a typical CF UME, data of the tip and substrate current–time behavior over the UME and surrounding glass, as well as the maps of substrate topography and surface charge, and FEM model details and simulations for the investigation of time response of the electrochemical cell (PDF)
Movie of *I*_sub_-*t* during the pulse delivery (AVI)
Movie of *I*_tip_-*t* during the pulse delivery (AVI)

## Supplementary Material

tg1c00006_si_001.pdf

tg1c00006_si_002.avi

tg1c00006_si_003.avi
