# Supplementary material for: Artificial Synapse: Spatiotemporal Heterogeneities in Dopamine Electrochemistry at a Carbon Fiber Ultramicroelectrode
Source: ACS Meas Sci Au. 2021 Jul 1;1(1):6–10. doi: 10.1021/acsmeasuresciau.1c00006 (PMC9836071; doi:10.1021/acsmeasuresciau.1c00006)
Supplement: Supplementary file 1 — tg1c00006_si_001.pdf [file tg1c00006_si_001.pdf]

Supporting Information

An Artificial Synapse: Spatiotemporal Heterogeneities in Dopamine Electrochemistry at a Carbon Fiber Ultramicroelectrode

Baoping Chen,[1] David Perry,[1] James Teahan,[1,2] Ian J. McPherson,[1] James Edmondson,[1,2] Minkyung Kang,[1] Dimitrios Valavanis,[1] Bruno G. Frenguelli[3] and Patrick R. Unwin[1,*]

[1]Department of Chemistry, [2]Molecular Analytical Science Centre for Doctoral Trainig, [3]School of Life Sciences, University of Warwick, Coventry, CV4 7AL, United Kingdom

*Corresponding author:

p.r.unwin@warwick.ac.uk

CONTENTS

**SI-1**	**Experimental Section**	**S3**
**SI-2**	**SICM Electrochemical Mapping**	**S6**
**SI-3**	**Voltammetric Electro-oxidation of Dopamine at a CF UME**	**S8**
**SI-4**	**Surface Characterization of a Typical CF UME**	**S8**
**SI-5**	**SICM Surface Charge Mapping**	**S9**
**SI-6**	**FEM Simulations**	**S11**
**SI-7**	**Time Response of the CF UME-Artificial Synapse Cell**	**S17**
**SI-8**	**Movies of I_{sub}-t and I_{tip}-t**	**S18**

SI-1 Experimental Section

Chemicals and Materials. All chemicals, of analytical grade, were purchased from Sigma-Aldrich and used as received. HEPES physiological saline was used as the bulk electrolyte, which contained 10 mM HEPES and 150 mM NaCl. The solution pH was adjusted to 7.4 with concentrated NaOH. All solutions were prepared with Milli-Q reagent grade water (resistivity ca. 18.2 MΩ cm at 25 °C) and filtered through a 20 nm filter (Anotop 25 syringe filter, Sigma-Aldrich) prior to use.

Nanopipettes and Electrodes. ~100 nm diameter nanopipettes were pulled from borosilicate glass capillaries (o.d. 1.2 mm, i.d. 0.69 mm, Harvard Apparatus) using a laser puller (P-2000, Sutter Instruments). The pulling parameters were: Line 1: Heat 385, Fil 3, Vel 30, Del 210, Pul –; Line 2: Heat 385, Fil 3, Vel 40, Del 170, Pul 120. Geometric characterization of the nanopipettes was performed using a JEOL ARM 200F scanning transmission electron microscope (STEM). A representative STEM image of the nanopipettes is shown in Figure S1. Two chloridized silver wires (Ag/AgCl) were used as the QRCEs, one in the nanopipette and the other in the bulk electrolyte. The equilibrium potential of the QRCE *vs* a saturated calomel electrode (SCE) was 45 mV in 50 mM KCl, 37 mV in 100 mM Dop^+ solution (as the chloride salt), and 22 mV in HEPES physiological saline. All potentials quoted in this work were with respect to the bulk QRCE unless noted otherwise.

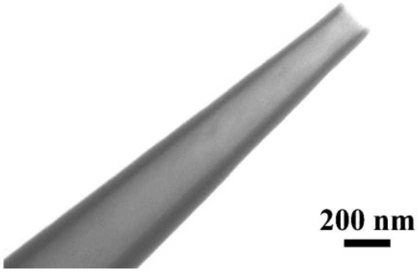

Figure S1. A representative STEM image of a nanopipette with ~100 nm inner diameter.

Preparation of CF UMEs. CF UMEs were fabricated according to an established procedure.[1] Briefly, a ~7 μm diameter CF (XAS grade, Goodfellow) was connected to a thin copper wire using conductive silver paint and inserted to the end of a borosilicate glass capillary. A seal between the glass and the CF was made using a temperature at approximately 85 °C under vacuum. To make an electrical contact, a larger copper wire was inserted into the capillary and connected to the thin copper wire with solder. Prior to use, the UME was polished with a micropipette polishing wheel with a diamond pad (diameter 0.1 μm, Buehler) and then an alumina slurry (diameter 0.05 μm, Buehler) on a soft microfiber polishing pad, followed by sonication in water to produce the finished surface.

Instrumentation. A picomotor (Newport, 8303 Picomotor Actuator) and a 15-μm-range single-axis piezoelectric positioner (Physik Instruments, P-753.1CD) were used for coarse and precise movement of the SICM nanopipette normal to the UME surface, respectively, while fine lateral movement of the UME was achieved by using a highly precise *XY* piezoelectric stage (Physik Instrumente, P-733.2DD) with a range of 30 μm on each axis. The SICM system was installed on an optical table (Newport, RS2000)

equipped with automatic leveling isolators (Newport, S-2000A-423.5). The current was measured using home-built current–voltage converters. Instrumentation control and data collection were conducted using custom-written LabVIEW (2017, National Instruments) code through an FPGA card (7852R, National Instruments). Data were acquired at a rate of 130 μs per point for the SICM electrochemical mapping (2 μs sampling time and averaging of 65 sample points).

Nanopipette Positioning Details. The nanopipette, filled with 100 mM Dop^+, as the chloride salt, was mounted on a custom-designed holder and positioned in the vicinity of the CF UME surface with a mechanical micropositioner (Newport, M-461-XYZ-M), aided by optical inspection using a 3MP digital camera (PixelLink, PL-B776U) with a 6× magnification lens. The surrounding electrochemical cell was then filled with HEPES physiological saline. To locate the CF UME with the nanopipette, an SICM hopping scan mode[2-4] was used to map the topography with a large scanning area of 30 μm × 30 μm and hopping steps of 1 μm. As a result of the polishing process, the CF surface was observed to be hundreds of nanometers higher than the surrounding glass sheath (see Figure S3 later) and so was readily found. During the topographical mapping, V_{tip} was biased at -80 mV, with respect to $QRCE_{bulk}$, to induce an ionic current for nanopipette vertical positioning and hold Dop^+ inside the nanopipette.

SI-2 SICM Electrochemical Mapping

The experimental setup has been described previously.[1] As shown in Figure S2a, the QRCE in bulk, denoted $QRCE_{bulk}$, was biased at $-V_{sub}$ relative to ground to control the electrochemical reaction at the UME surface (at ground). The resulting current through the UME is denoted I_{sub}. An ionic current, I_{tip}, between the two QRCEs was generated by a bias of V_{tip}, relative to $QRCE_{bulk}$. I_{sub} and I_{tip} were measured throughout the entire imaging process. The mapping protocol ultilized hopping mode SICM[2-4] in a raster scanning pattern, starting at the top left of images and scanning down vertically. Traces of the tip *z*-position and V_{tip} at each pixel are shown in Figure S2b, with the different time periods (I, II, III, IV) corresponding to the features in Figure 1 in the main manuscript.

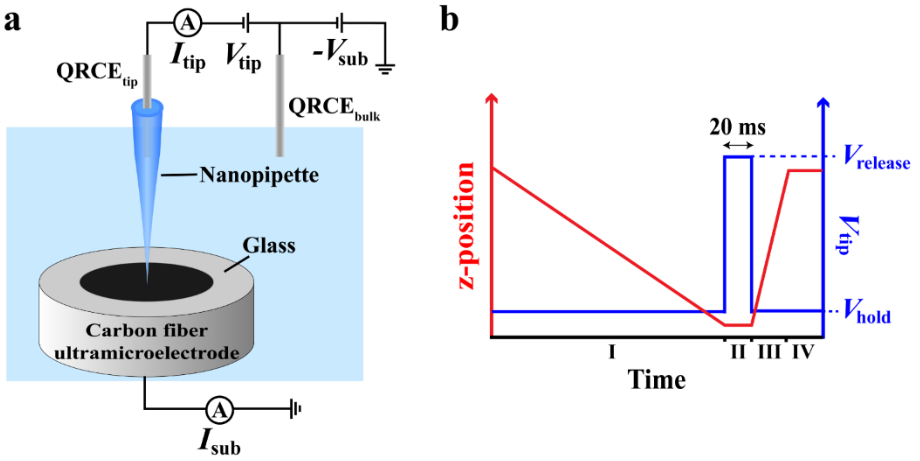

Figure S2. (a) Schematic of the experimental setup. (b) A trace of tip *z*-position and V_{tip} at each pixel in the mapping protocol.

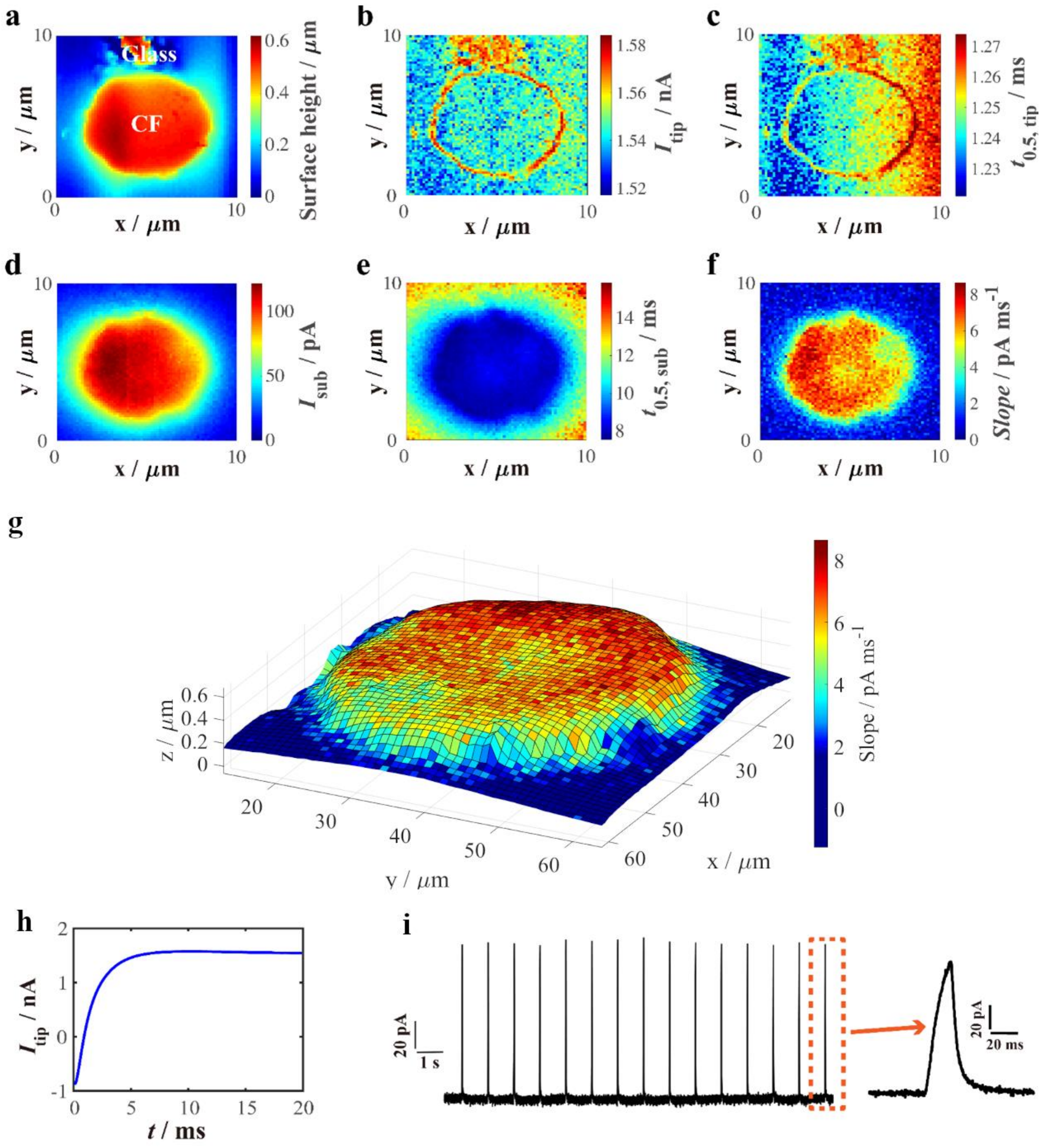

Figure S3. Images of (a) topography, (b) I_{tip} at the end of the Dop^+ pulse release, (c) time of I_{tip} to reach half the final magnitude change, (d) final value of I_{sub} for each Dop^+ release, (e) time for I_{sub} to reach half the final value, and (f) rate of increase of I_{sub} at a time of 2.5 ms after initiation of the pulse. (g) cropped data from (f) plotted on the 3D topography from (a). (h) A typical I_{tip}-t transient, and (i) I_{sub}-t transients at a series of pixels when the tip was over the CF UME surface, with a representative current spike depicted. All the figures were extracted within the same 10 μm × 10 μm area scan. Step size between pixels: 150 nm. Further explanations are given in the main manuscript.

SI-3 Voltammetric Electro-oxidation of Dopamine at a CF UME

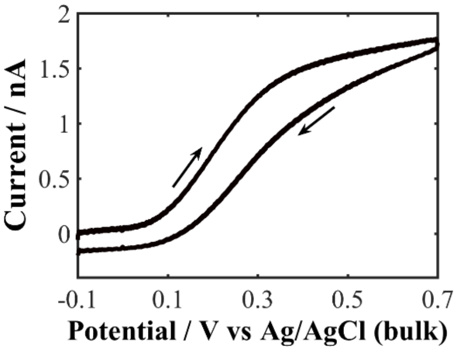

Figure S4. Voltammetry of a typical CF UME obtained in a bulk solution containing 1 mM dopamine hydrochloride with 150 mM NaCl and 10 mM HEPES (pH 7.4). The scan rate was 100 mV s^{-1}.

SI-4 Surface Characterization of a Typical CF UME

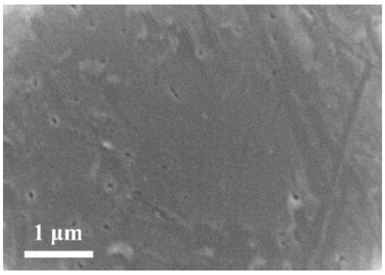

Figure S5. A representative field emission-scanning electron microscopy (FE-SEM) image of an area of a typical CF UME surface.

SI-5 SICM Surface Charge Mapping

An established SICM hopping scan mode[5] was used with the same experimental setup as shown in Figure S2a to elucidate information about the surface charge of the CF UME surface. As before, scanning simultaneously captures the topography, allowing alignment of datasets from separate experiments (Figure S7a) and analysis of surface roughness (Figure S7b). In the experiment, a ~100 nm diameter nanopipette was used as the tip. 50 mM KCl was used as both the tip and bulk electrolyte. Two Ag/AgCl electrodes were used as the QRCEs, one in the tip ($QRCE_{tip}$) and the other in the bulk solution ($QRCE_{bulk}$). The regime for each pixel was as follows (Figure S6): (I) The tip was translated towards the UME surface with V_{tip} biased at 40 mV with respect to $QRCE_{bulk}$. When the ionic current (I_{tip}) between the two QRCEs reduced by a chosen percentage threshold of 2.5%, the tip stopped approaching. (II) A 20 ms pulse of V_{tip} to -400 mV was then applied. (III) V_{tip} was stepped back to 40 mV as the tip was retracted by 2 μm to the bulk. (IV) A second 20 ms pulse of V_{tip} to -400 mV was performed in the bulk solution. (V) V_{tip} was stepped to 40 mV and the UME was then moved to the next pixel.

During SICM surface charge mapping, the UME was held at a constant potential value of 0.7 V (V_{sub} = 0.7 V, relative to $QRCE_{bulk}$), and I_{tip} was continuously measured throughout. A normalized I_{tip} map produced by taking an average of the final few points of the I_{tip}-t curve with the tip near the surface divided by an average of the final few points of bulk I_{tip}-t curve at each pixel (Figure S7c) was used to elucidate surface charge.[5] By using FEM simulations (section SI-6), the experimental current map was

converted to values of the surface charge, as shown in Figure S7d.

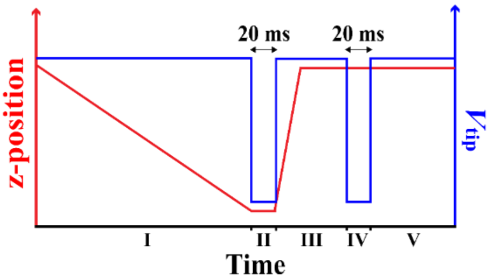

Figure S6. A trace of *z*-position and V_{tip} applied at each pixel in the SICM surface charge mapping protocol.

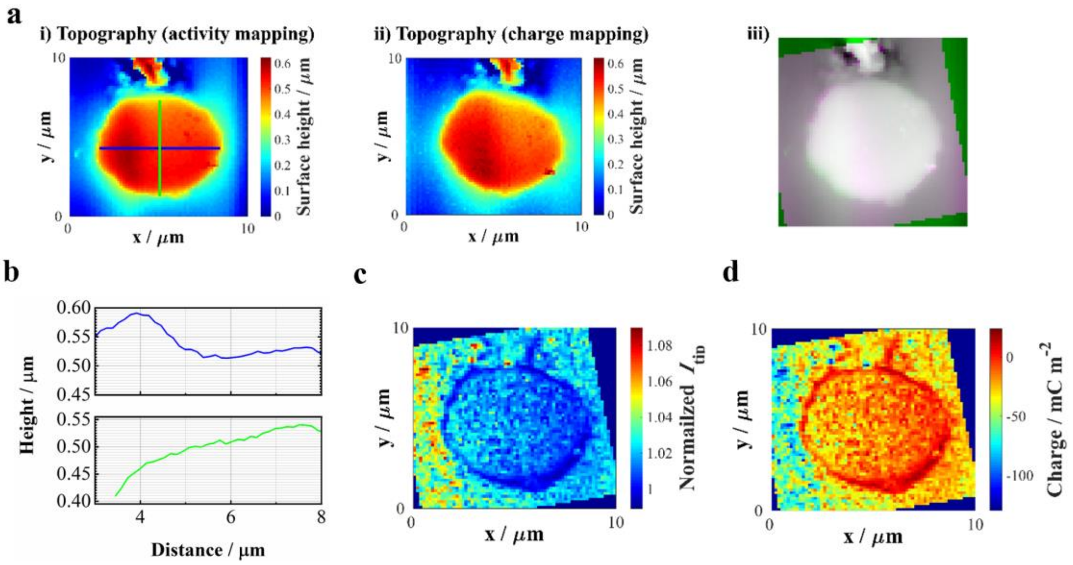

Figure S7. (a) Topography maps from (i) UME activity and (ii) charge mapping are aligned to allow comparison between data from different experiments. A fragment of glass (also apparent in Figure S3a,b,c) from the polishing procedure (SI-1) was advantageous for (iii) aligning maps (i) and (ii) to allow identical co-location analysis of charge and activity data. (b) Line profiles along lines indicated on (a)(i) showing typical surface roughness. (c) Normalized tip current map from charge mapping experiments, analyzed as described in the text to produce (d) a quantified surface charge image. Following the alignment process in (a)(iii), this could be compared at the nanoscale with the electrochemical activity maps in Figure S3 (d). The quantified surface charge image was generated through FEM simulations (Section SI-6). Step size between pixels: 150 nm.

SI-6 FEM Simulations

FEM simulations were constructed in COMSOL Multiphysics (v5.4), which followed previous work.[1,5] A schematic of the FEM simulation domain is shown in Figure S8a.

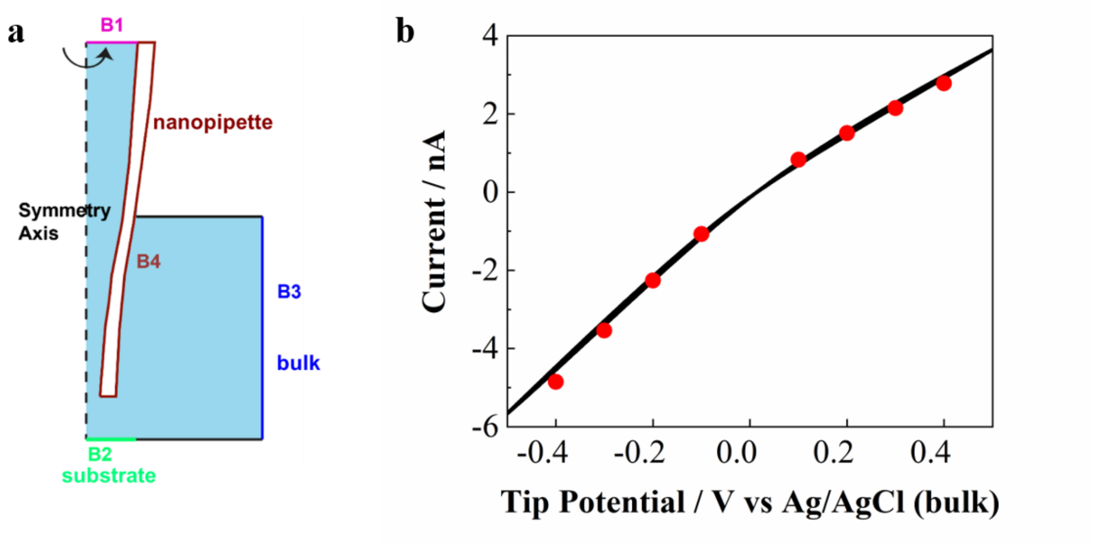

Figure S8 (a) Schematic of the FEM simulation domain with significant boundaries labelled (not to scale). (b) Experimental current-potential curve of the nanopipette under activity mapping conditions (lines) and the simulated steady-state current-potential behavior (points).

Typical dimensions of the nanopipettes used were extracted from STEM images and used to construct the geometry of the nanopipette simulation domain. All tip potentials were applied to the upper nanopipette boundary, labelled B1. B3 was held at ground in all simulations. B4 had a surface charge density of -40 mC m^{-2} applied in all simulations, estimated from previous work.[6] This value is justified by the resulting close agreement between the experimental and simulated current-potential curves (Fig S8b), with the rectification behavior (enhanced current at negative potentials,

decreased current at positive potentials) supporting the conclusion of negative wall charge under these conditions.[7] For the simulations corresponding to the surface charge mapping experiment, the concentration at both B1 and B3 (and the corresponding bulk phase) was maintained at 50 mM K^+ and 50 mM Cl^-. Variable surface charge densities were applied to boundary B2. For the simulations corresponding to the electrochemical mapping experiment, the concentration at B1 was maintained at 100 mM Dop^+ and 100 mM Cl^-, representing bulk solution in the nanopipette, whilst a concentration of 150 mM Na^+ and 150 mM Cl^- at B3 was maintained, considered to be the bulk in the bath. For computation efficiency, the HEPES, as a very minor species (10 mM) with organic anions of low mobility,[8] was neglected. All conditions relating to the electro-oxidation of dopamine were applied to B2.

The electrostatics, transport of diluted species and laminar flow modules of COMSOL Multiphysics were used to model the experimental system. In all simulations, ion transport was assumed to follow the Nernst-Planck equation, where the flux J_i of species i is given as:

$$J_i = -D_i \nabla c_i - z_i \frac{F}{RT} D_i c_i \nabla \phi + c_i u \quad \text{(S1)}$$

where D_i, z_i, and c_i are the diffusion coefficients, charge number and concentrations of species i, respectively. u is the solution velocity described below (eq. S3). Ion diffusion coefficients were taken from the CRC handbook.[9] The infinite dilution values of K^+ (1.96×10^{-5} cm^2 s^{-1}), Na^+ (1.2×10^{-5} cm^2 s^{-1}), and Cl^- (2.05×10^{-5} cm^2 s^{-1}) were used, which is considered reasonable, as we employed a self-referencing method for surface

charge mapping and for electrochemical mapping the diffusion coefficient and mobility of Dop^+ is most important. The diffusion coefficient for Dop^+ was taken from the literature value to be 0.6×10^{-5} cm^2 s^{-1}.[10] F, R and T are the Faraday constant, gas constant and absolute temperature, respectively. ϕ is the electric potential described by the Poisson equation (eq. S2):

$$\nabla^2\phi = -\frac{F}{\varepsilon\varepsilon_0}\sum_i z_i c_i \quad \text{(S2)}$$

where ε is the dielectric constant of the solution and ε_0 is the vacuum permittivity. The coupling of eq. S1 and eq. S2 results in the formation of a diffuse double layer at charged surfaces. The solution velocity was described by the incompressible Navier-Stokes equation with electroosmotic flow (EOF) incorporated (eq. S3):

$$u\nabla u = \frac{1}{\rho}\left(-\nabla p + \mu\nabla^2 u - F\left(\sum_i z_i c_i\right)\nabla\phi\right) \quad \text{(S3)}$$

where ρ is the solution density, u is the solution velocity and p is the pressure.

FEM simulations were first employed to estimate the experimental working distances for the surface charge mapping experiment and the electrochemical mapping experiment, by performing steady-state simulations at the respective holding potential at different tip-substrate separations.[1, 5] The obtained simulated approach curves are shown in Figure S9. These yielded a working distance of ca. 25 nm corresponding to the experimental feedback threshold of 2.5% for the surface charge mapping experiment and ca. 37 nm corresponding to the experimental feedback threshold of 2% for the electrochemical mapping experiment.

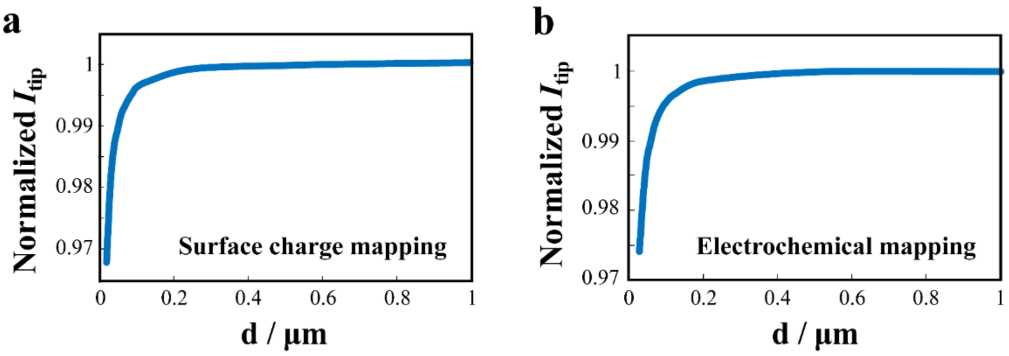

Figure S9. FEM simulations of normalized tip current (steady-state tip current at distance, *d*, from the surface divided by the steady-state tip current in bulk) versus tip-substrate distance for: (a) surface charge mapping, where a tip-substrate separation of ca. 25 nm corresponds to the experimental feedback threshold of 2.5%; and (b) SICM electrochemical mapping, generating a tip-substrate separation of ca. 37 nm corresponding to the experimental feedback threshold of 2%.

FEM simulations were then employed to quantify the surface charge density of the CF UME, following earlier work.[5] Time-dependent simulations were performed with different values of surface charge density applied to boundary B2. The initial condition for each time-dependent simulation was a steady-state simulation which had been performed at the holding potential (40 mV). For the time-dependent simulations, the potential applied to B1 was jumped to -400 mV. Simulations were performed at a separation of 25 nm as in the experiments, and 2 µm to represent bulk solution. Figure S10 shows a working curve of normalized I_{tip} against surface charge density and these data were used to convert the experimental current data (Figure S7b) to surface charge densities (Figure S7c).

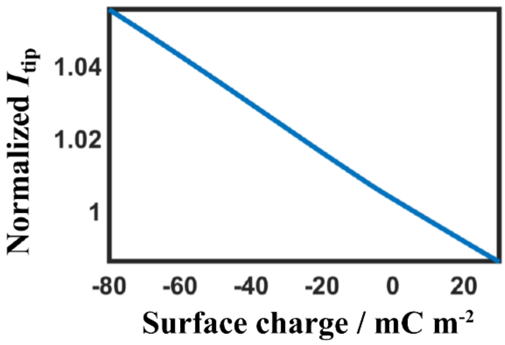

Figure S10. Working curve of normalized I_{tip} against surface charge density used to convert experimental current data to surface charge densitities shown in Figure S7c.

Simulated I_{tip}-t and I_{sub}-t transients upon the release of Dop^+ for the electrochemical mapping experiment could be produced (as shown in Figure S12 later), by performing time-dependent simulations at the working distance of 37 nm. Based on the fact that the electro-oxidation was relatively driven and at only one applied potential (amperometry), we considered a first-order process in the near-interface concentration of Dop^+, as shown in eq. S4, with a concomitant inwards flux of Dopaminequinone (DOQ^+) and $2H^+$.

$$J_{Dop^+} = -k'c_{Dop^+} \tag{S4}$$

where k' is the reaction rate constant, and c_{Dop^+} pertains to the near-interface concentration of Dop^+. This condition relating to Dop^+ electro-oxidation was applied to boundary B2 and a two-electron transfer oxidation was considered. The substrate current was calculated by integrating the local current density across the electrode surface. The tip current was calculated as the rate of charge flow passing a boundary spanning the inside of the nanopipette, following previous work.[1,5,11] The initial

condition for the time-dependent simulation was a steady-state simulation which had been performed at the holding potential (-80 mV).

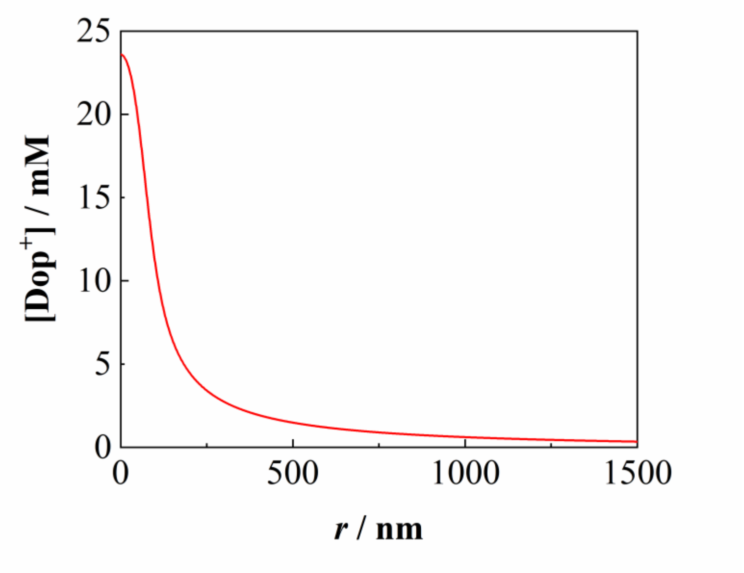

Figure S11. Surface dopamine concentration at the end of the delivery pulse as a function of distance, *r*, from the cylindrical axis of the nanopipette.

Figure 2e in the paper showed that Dop^+ delivered to the surface was concentrated on a region of the surface of approximately the tip size. This is quantified in Figure S11 by the corresponding plot of $[Dop^+]$ in the near CF UME region at the end of the pulse as a function of radial distance, *r*, from the cylindrical axis of the nanopipette. The effects of lateral diffusion are minimized (and hence resolution maximized) at short times after the pulse, as seen from the increased contrast observed in Figure 2d (data from 2.5 ms after the pulse) compared to Figure 2b (data from 20 ms after the pulse) , and the change in spatial contrast in activity Movie S1 as time progresses.

SI-7 Time Response of the CF UME-Artificial Synapse Cell

When a potential pulse is applied to the SICM tip, there is a response time due to the resistance and capacitance of the two electrode cell, dominated by the tip resistance. The effect is to introduce a lag in the application of the pulse potential for the delivery of Dop^+ from the tip to the CF UME surface in the electrochemical mapping experiment, thus leading to a short delay at the start of each I_{sub}-t curve, as observed in Figure 2a. This can be estimated using FEM simulations and the familiar *RC* circuit diagram, where the behavior of the tip current-time (*i*-*t*) transient when applying a potential step of magnitude *E* (280 mV used, from -80 mV to 200 mV) is described as:[12]

$$i = \frac{E}{R} e^{-t/RC} \quad \text{(S5)}$$

where *R* and *C* are the resistance and capacitance of the system, respectively. The effect of the *RC* constant was estimated by filtering the potential pulse function with a first-order low pass finite impulse response filter with cut-off frequency $f_0 = 1 / (2\pi \cdot \tau)$, where $\tau = RC$ prior to applying it in the simulation. Processing was carried out in Matlab using the designfilt function.[13, 14] Figure S12a shows simulated I_{tip}-t transients with (and without) this treatment (eq. S5). It is clear that by varing *R* and *C* in eq. S5, the simulated tip current response is able to resemble the experimental current transient at a meausured value of R = 108 MΩ and an estimated, but reasonable, value of C = 20 pF (which includes stray capacitance).[15]

The corresponding simulated I_{sub}-t transient could be produced, by performing time-dependent simulations at a working distance of 37 nm, by varying k' in eq. S4 representing the electro-oxidation of Dop^+ at electrode surface (boundary B2), until the simulated current transient resembled the experimental response ($k' = 2.5 \times 10^{-3}$ cm s^{-1} giving the optimal fit). It is observed from Figure S12b that the simulated I_{sub}-t curve,

taking account of the *RC* time constant of the setup, has the same general shape as the experimentally recorded data. A corresponding simulated concentration profile of Dop^+ at the end of the pulse release was also extracted and shown in Figure 2e in the main manuscript. The effect of large resistance of the tip is negligible at the end of pulse release, where a steady-state delivery is obtained, as observed from the experimental I_{tip}-t transient in Figure S12a.

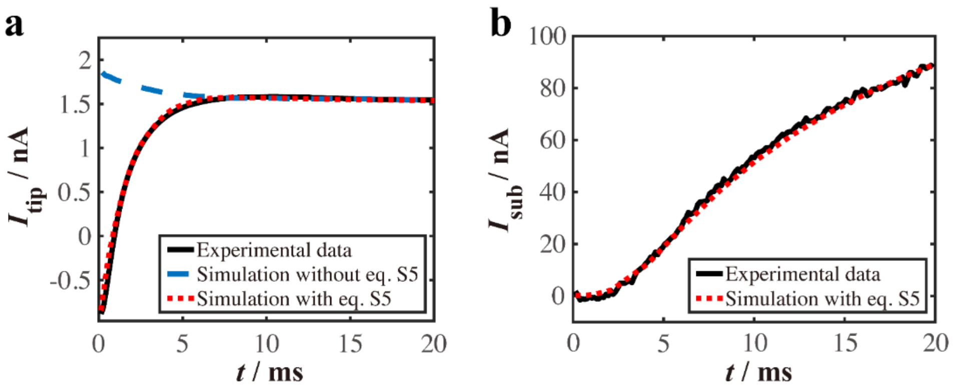

Figure S12. (a) Comparision of the simulated I_{tip}-t transients (with/without the potential step filtered based on eq. S5) along with the experimental data. (b) The corresponding experimental and simulated (with filtered potential step) I_{sub}-t transients.

SI-8 Movies of I_{sub}-t and I_{tip}-t

Movies of I_{sub} and I_{tip} as a function of time during the pulse delivery process are provided in Movie S1 and Movie S2, respectively, which are corresponding to the data in Figure S3 and Figure 2 of the manuscript.

REFERENCES

1. Chen, B.; Perry, D.; Page, A.; Kang, M.; Unwin, P. R., Scanning Ion Conductance Microscopy: Quantitative Nanopipette Delivery–Substrate Electrode Collection Measurements and Mapping. *Anal. Chem.* **2019,** *91* (3), 2516-2524.
2. Chen, C.-C.; Zhou, Y.; Baker, L. A., Scanning ion conductance microscopy. *Ann. Rev. Anal. Chem.* **2012,** *5*, 207-228.
3. Zhang, Y.; Takahashi, Y.; Hong, S. P.; Liu, F.; Bednarska, J.; Goff, P. S.; Novak, P.; Shevchuk, A.; Gopal, S.; Barozzi, I., High-resolution label-free 3D mapping of extracellular pH of single living cells. *Nature Comm.* **2019,** *10* (1), 1-9.
4. Happel, P.; Dietzel, I. D., Backstep scanning ion conductance microscopy as a tool for long term investigation of single living cells. *J. Nanobiotech.* **2009,** *7* (1), 7.
5. Page, A.; Perry, D.; Young, P.; Mitchell, D.; Frenguelli, B. G.; Unwin, P. R., Fast nanoscale surface charge mapping with pulsed-potential scanning ion conductance microscopy. *Anal. Chem.* **2016,** *88* (22), 10854-10859.
6. Perry, D.; Momotenko, D.; Lazenby, R. A.; Kang, M.; Unwin, P. R., Characterization of nanopipettes. *Anal. Chem.* **2016,** *88* (10), 5523-5530.
7. Wei, C.; Bard, A. J.; Feldberg, S. W., Current Rectification at Quartz Nanopipet Electrodes. *Anal. Chem.* **1997,** *69* (22), 4627-4633.
8. Chen, S.-H.; Chao, A.; Tsai, C.-L.; Sue, S.-C.; Lin, C.-Y.; Lee, Y.-Z.; Hung, Y.-L.; Chao, A.-S.; Cheng, A.-J.; Wang, H.-S.; Wang, T.-H., Utilization of HEPES for Enhancing Protein Transfection into Mammalian Cells. *Molecular Therapy - Methods & Clinical Development* **2019,** *13*, 99-111.
9. Lide, D. R., *CRC Handbook of Chemistry and Physics*. CRC press: 2004; Vol. 85.
10. Gerhardt, G.; Adams, R. N., Determination of diffusion coefficients by flow injection analysis. *Anal. Chem.* **1982,** *54* (14), 2618-2620.
11. McKelvey, K.; Kinnear, S. L.; Perry, D.; Momotenko, D.; Unwin, P. R., Surface charge mapping with a nanopipette. *J. Am. Chem. Soc.* **2014,** *136* (39), 13735-13744.
12. Bard, A. J.; Faulkner, L. R.; *Electrochemical methods,* Wiley New York: 1980, 1st edition.
13. Chen, C.-H.; Ravenhill, E. R.; Momotenko, D.; Kim, Y.-R.; Lai, S. C. S.; Unwin, P. R., Impact of Surface Chemistry on Nanoparticle–Electrode Interactions in

the Electrochemical Detection of Nanoparticle Collisions. *Langmuir* **2015,** *31* (43), 11932-11942.

14. Robinson, D. A.; Edwards, M. A.; Ren, H.; White, H. S., Effects of Instrumental Filters on Electrochemical Measurement of Single-Nanoparticle Collision Dynamics. *ChemElectroChem* **2018,** *5* (20), 3059-3067.

15. McKelvey, K.; Perry, D.; Byers, J. C.; Colburn, A. W.; Unwin, P. R., Bias modulated scanning ion conductance microscopy. *Anal. Chem.* **2014,** *86* (7), 3639-3646.
